# From Twin Blocks to Quad Blocks: A Two-Phase Approach to Class II Correction

**DOI:** 10.7759/cureus.55036

**Published:** 2024-02-27

**Authors:** Lovely Bharti, Abhishek D Sanchla, Pallavi Daigavane, Sunita Shrivastav, Ranjit Kamble, Aksha Bhargava, Shourya Bharadwaj, Aathira Surendran, Dhwani Suchak

**Affiliations:** 1 Orthodontics and Dentofacial Orthopedics, Datta Meghe Institute of Higher Education and Research, Wardha, IND; 2 Oral and Maxillofacial Surgery, Mahatma Gandhi Dental College and Hospital, Jaipur, IND; 3 Orthodontics, Jaipur Dental College, Jaipur, IND

**Keywords:** twin block, orthopedic effect, functional appliance, skeletal class ii division 1 malocclusion, quad block

## Abstract

Twin Block, a functional orthodontic appliance, has a major impact on the dentoalveolar structures with limited skeletal effects. In specific clinical scenarios, particularly in growing patients such as class II division 1 where the mandible is retropositioned, these appliances can effectively address the malocclusions. Patient compliance plays a crucial role in the success of these appliances, which also streamline subsequent phases of fixed appliance treatment. In the current case report, a Twin Block appliance followed by a Quad Block was given to a 12-year-old boy to refine the occlusion. The design and treatment outcomes of the appliance are discussed in this case study.

## Introduction

Orthodontic treatment aims to correct malocclusions and enhance facial aesthetics, oral function, and overall oral health. Class II malocclusion, characterized by a discrepancy between the sizes of the upper and lower jaws resulting in an anterior overjet, is a common orthodontic concern. Patients with class II malocclusion often present with various associated issues, such as lip incompetence, deep overbite, and dental crowding, which can impact both aesthetics and function [1].

This case report presents the orthodontic treatment of a patient with class II malocclusion, focusing on the use of functional appliances followed by fixed appliances to address the presenting concerns. The patient's chief complaint of forwardly placed teeth and inability to close the lips was indicative of the severity of the malocclusion and the need for intervention.

Orthodontic treatment planning requires careful consideration of various factors, including the patient's skeletal and dental characteristics, treatment goals, patient compliance, and preferences. In this case, the treatment plan was designed to address the patient's specific malocclusion issues while considering the potential advantages and drawbacks of different treatment modalities [2].

The introduction of functional appliances, such as the Clark Twin Block appliance, aimed to achieve early correction of the malocclusion, reduce overjet and overbite, and establish a class I molar relationship. Subsequent treatment phases involved fixed appliances to further refine the occlusion, align the dental arches, and address any remaining dental crowding [3,4].

The Quad Block appliance is an advancement in functional orthodontics, featuring four strategically placed bite blocks in both the maxillary and mandibular arches. By engaging the occlusal surfaces of molars and premolars, it promotes coordinated mandibular advancement and bite correction. This innovative appliance effectively addresses class II malocclusions by redistributing forces and stimulating mandibular growth, enhancing treatment outcomes with improved patient comfort and compliance [5,6].

This case report illustrates the importance of a phased approach to orthodontic treatment, emphasizing the role of functional appliances in early intervention and the subsequent use of fixed appliances to achieve optimal outcomes. By documenting the treatment process and outcomes in detail, this report aims to contribute to the existing body of knowledge in orthodontics and provide insights for orthodontic practitioners facing similar clinical challenges.

## Case presentation

A 12-year-old patient reported to the Department of Orthodontics with a chief complaint of forwardly placed teeth and unable to close the lips. Upon initial examination, the patient presented with a class II malocclusion with a prominent overjet of 6 mm and a deep overbite. He displayed a convex profile with a retruded mandibular position. His dental arches were moderately crowded, particularly in the lower anterior region.

**Figure 1:**
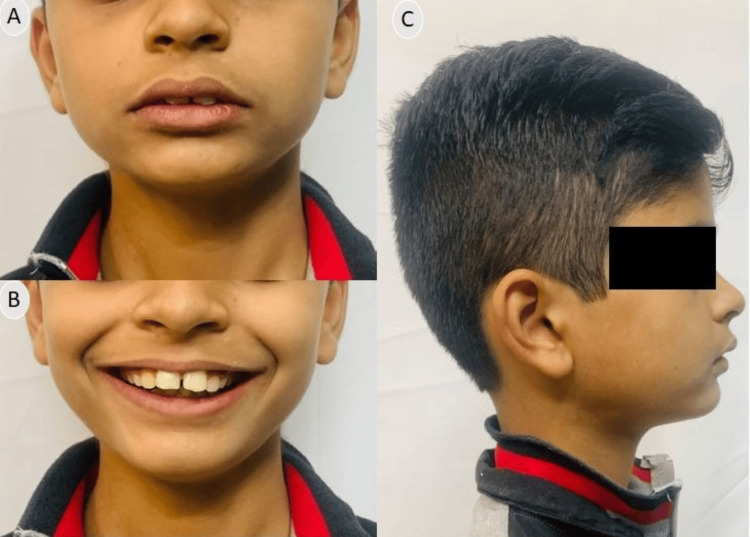
Pre-treatment extraoral photographs (A) Frontal; (B) Smiling; (C) Profile

Clinical examination revealed a minor class II skeletal pattern with an average Frankfort mandibular plane angle (FMPA) and lower anterior face height. Facial symmetry was observed, but the patient exhibited lip incompetence, characterized by the lower lip resting behind the upper central incisors (Figure 1). There was moderate calculus present on lower anteriors, thus it required enhancement prior to initiating orthodontic treatment.

**Figure 2:**
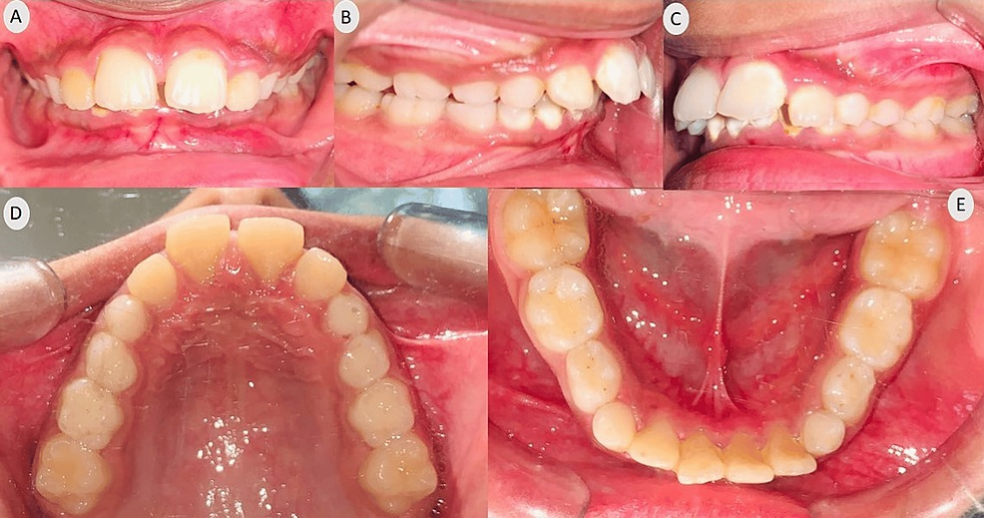
Pre-treatment intraoral photographs (A) Frontal; (B) Right lateral; (C) Left lateral; (D) Maxillary occlusal; (E) Mandibular occlusal

Upon intra-oral examination, all incisors and first molars were present in both arches, along with deciduous canines and deciduous first and second molars bilaterally. The upper arch showed spacing with a midline diastema, and there was minor crowding in the lower labial area (2.5 mm). The incisor relationship was classified as class II division 1, with an overjet of 6 mm and an increased overbite extending completely to the palate. Centrelines were found to be coincident (Figure 2).

**Figure 3:**
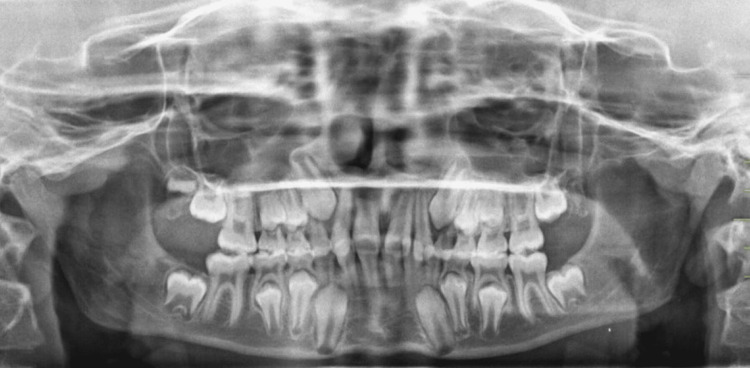
Pre-treatment orthopantomogram (OPG)

**Figure 4:**
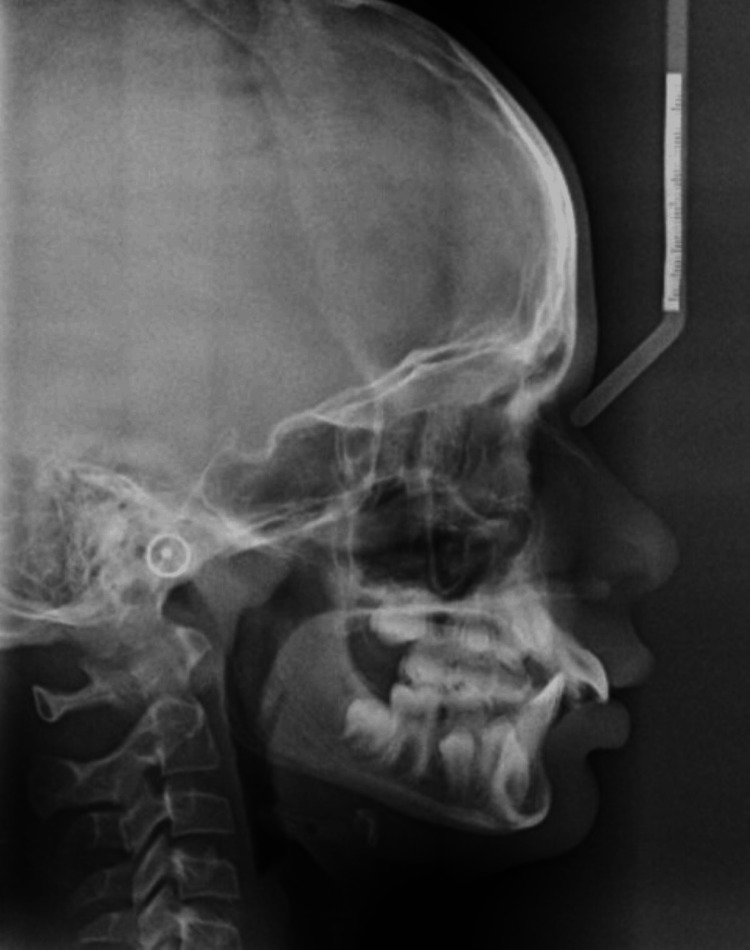
Pre-treatment lateral cephalogram

In the panoramic X-ray (orthopantomogram (OPG)), all permanent teeth were present including the developing upper right third molar, premolars had not erupted yet in all quadrants, and deciduous canines and molars were present but no signs of developing upper left, lower right, or left third molars were visible at this stage (Figure 3). The cephalometric assessment revealed a mild class II skeletal pattern with an ANB (Point A, Nasion, and Point B) value of 5° (Table 1). The interincisal angle was 117°, with both the lower incisor to Apo (Point A to Pogonion) and lower lip to E (Ricketts Esthetic) line being within normal limits (Figure 4, Table 1).

**Table 1:** Pre- and post-treatment cephalometric values SNA: Sella nasion point A; SNB: Sella nasion point B; ANB: Point A nasion point B; FMA: Frankfort mandibular plane angle; UI: Upper incisor; LI: Lower incisor; IMPA: Incisor mandibular plane angle; E: Esthetic line

Variables	Mean	Pre-treatment	Post-phase II treatment	Difference
Maxilla to cranium				
SNA (degree)	82±2	82	84	2
Mandible to cranium				
SNB (degree)	80±2	77	80	3
Maxilla to mandible				
ANB (degree)	2±2	5	4	1
Vertical relationship				
Y-axis angle (degree)	53–66	57	60	3
Facial axis angle (degree)	90	92	95	3
FMA angle (degree)	25	26	26	0
Maxillary dental				
UI to NA (angle)	22	34	27	7
UI to NA (mm)	4	8	5	3
UI to SN (angle)	102±2	118	115	3
Mandibular dental				
LI to NB (angle)	25	22	27	5
LI to NB (mm)	4	7	5	2
IMPA (angle)	90±5	95	95	0
Maxilla to mandible (dental)				
UI to LI (angle)	130	117	120	3
Soft tissue analysis				
Nasomental angle	120-132	119	109	10
Nasolabial angle (degree)	102±4	101	102	1
Upper lip prominence (mm)	1-2	4.5	3	1.5
Lower lip prominence (mm)	1	2.5	0	2.5
E-line (upper lip/lower lip)(mm)	-4/-2	2/1	0/3	2/2
Soft tissue chin thickness (mm)	10-12	11	12	1

The primary goals included in phase I of the treatment were advancing the mandible and enhancing the profile, decreasing both the overbite and overjet, and establishing a class I molar relationship while securing anchorage.

The objectives in phase II of the treatment were maintaining the class I molar relationship and continuing mandibular advancement, resolving lower arch crowding, aligning and leveling the arches, and closing the space in the upper labial segment.

The upper buccal segment was proposed to be distalized with a headgear appliance to correct the molar relationship and create space, thereby reducing overjet. Furthermore, the headgear treatment was considered beneficial due to its restraining effect on the maxilla [7]. However, when presented with both appliance options, the patient expressed a preference for the Twin Block appliance over the headgear.

While some argue that this case could be achieved solely with fixed appliances using class II intermaxillary traction, it's worth noting the significant drawbacks of this approach. Such treatment might lead to lower incisor proclination and could pose challenges in achieving a class I molar correction. Moreover, ensuring adequate anchorage becomes crucial, as any loss of anchorage due to mesial movement of the upper molars could complicate efforts to reduce overjet and correct the molar relationship.

Fabrication of appliances

Fabrication of Twin Block and Quad Block involved construction bite, which was taken with modeling wax with sagittal advancement of 7 mm and 3 mm of vertical opening. After this, the construction bite was secured with upper and lower working cast models, and articulation was done using a mean value articulator. The construction bite was then removed from the mounted cast and a cold mould seal was applied all over the occlusal and tissue surface of working cast models, wire component (delta clasp, labial bow, and ball end clasp) was then secured in the cast model, and bite blocks were prepared in both the upper arch and lower arch at 70° angulation using cold cure acrylic resin. All steps of fabrication of Quad Block were the same as of Twin Block except attachment of molar tubes (0.022") on all four acrylic bite blocks in the dough stage of cold cure acrylic resin.

**Figure 5:**
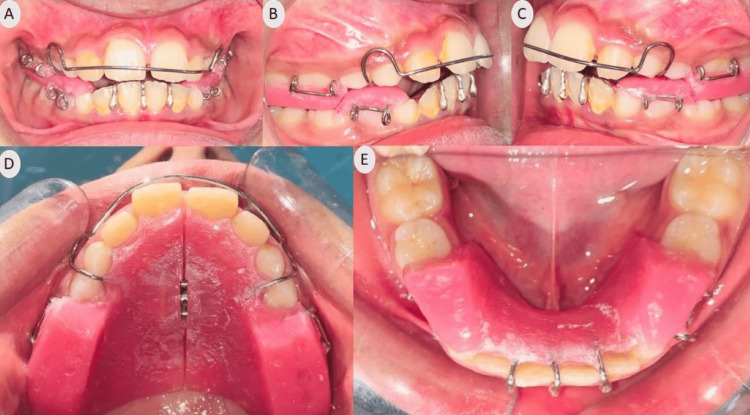
Intraoral photographs with Twin Block appliance (A) Frontal view; (B) Right lateral view; (C) Left lateral view; (D) Maxillary occlusal view; (E) Mandibular occlusal view

The upper component of the Twin Block (70° angulation) consisted of an acrylic baseplate with an expansion screw in the midline that covered the palate and occlusal surfaces of the first and second deciduous molars, as well as an inclined plane at the acrylic block's mesial end (Figure 5). A labial bow was used to ensure anterior retention of the appliance (Figure 5D).

The lower component of the appliance comprised left and right blocks inclined at 70° and three ball end clasp, which was placed in the interdental spaces of central and lateral incisors. Both components were equipped with delta clasps on the second deciduous molar in the upper arch and the first deciduous molar in the lower arch to ensure retention for posteriors (Figure 5). As there was crowding in the lower anteriors, incisal capping with acrylic could not be done to prevent lower incisor proclination and thus resulted in the flaring of incisors.

Management of appliances

Bite blocks were checked for comfortable occlusion, and appointments were scheduled every three to four weeks. Trimming of the appliance was initiated once pterygoid response (a new pattern of muscle behavior) was achieved, which approximately took 45-50 days. Once it was achieved, trimming of bite blocks 1-2 mm per month was done to allow eruption of molars.

Treatment progress

Phase I of the treatment involved using a functional appliance, specifically the Clark Twin Block appliance, to reduce overjet, achieve class I molar relationships, and establish anchorage at the start of treatment to streamline the fixed appliance stage. Furthermore, inducing minor skeletal changes could improve the patient's profile.

**Figure 6:**
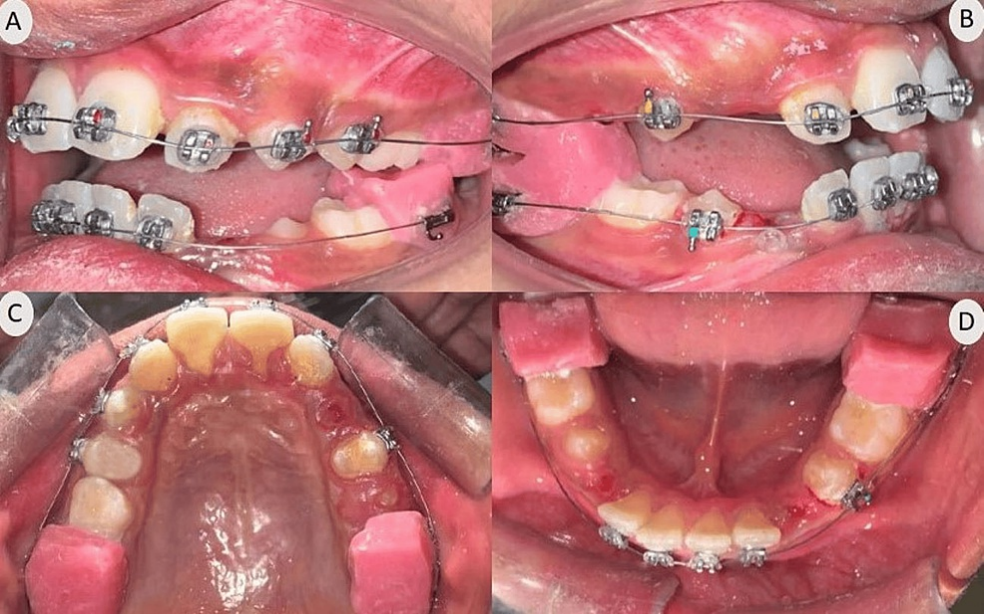
Intraoral photographs with Quad Block appliance (A) Left lateral view; (B) Right lateral view; (C) Maxillary occlusal view; (D) Mandibular occlusal view

Subsequently, in phase II, Quad Block was introduced to provide additional posterior bite blocks in both the maxillary and mandibular arches, facilitating continued mandibular advancement and bite correction (Figure 6). Following this, upper and lower fixed appliances (0.022′′ slot brackets) were utilized along with 0.016 nickel-titanium wire to address tasks such as space closure, leveling, and alignment.

**Figure 7:**
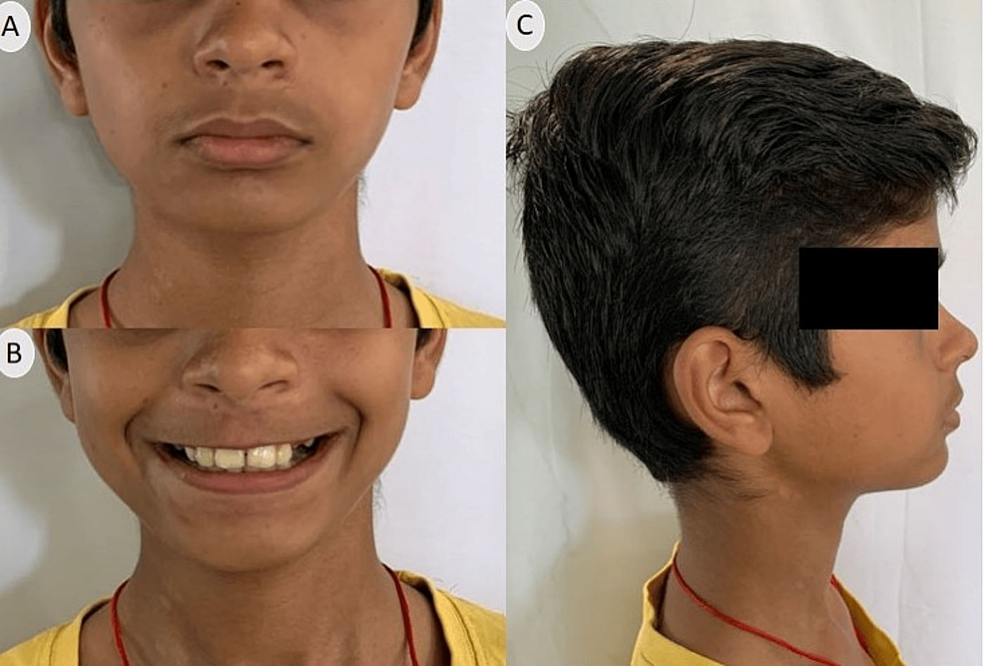
Extraoral photographs after Twin Block therapy (A) Frontal; (B) Smiling; (C) Profile

Post-phase I treatment results can be appreciated with the extraoral photographs, which show competent lips with a straight profile of the patient achieved following Twin Block therapy (Figure 7).

**Figure 8:**
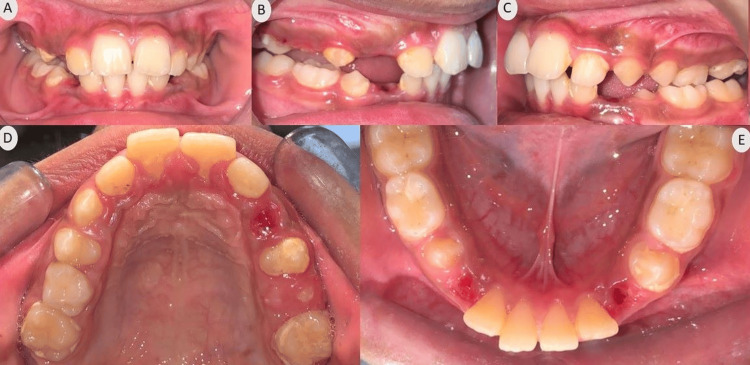
Intraoral photographs after Twin Block therapy (A) Frontal view; (B) Right lateral view; (C) Left lateral view; (D) Maxillary occlusal view; (E) Mandibular occlusal view

Intraoral photographs post-Twin Block therapy show reduced overjet and overbite and class I molar relationship along with erupting cuspid on the left upper quadrant. There was mild crossbite observed after Twin Block therapy in the left quadrant (Figure 8).

**Figure 9:**
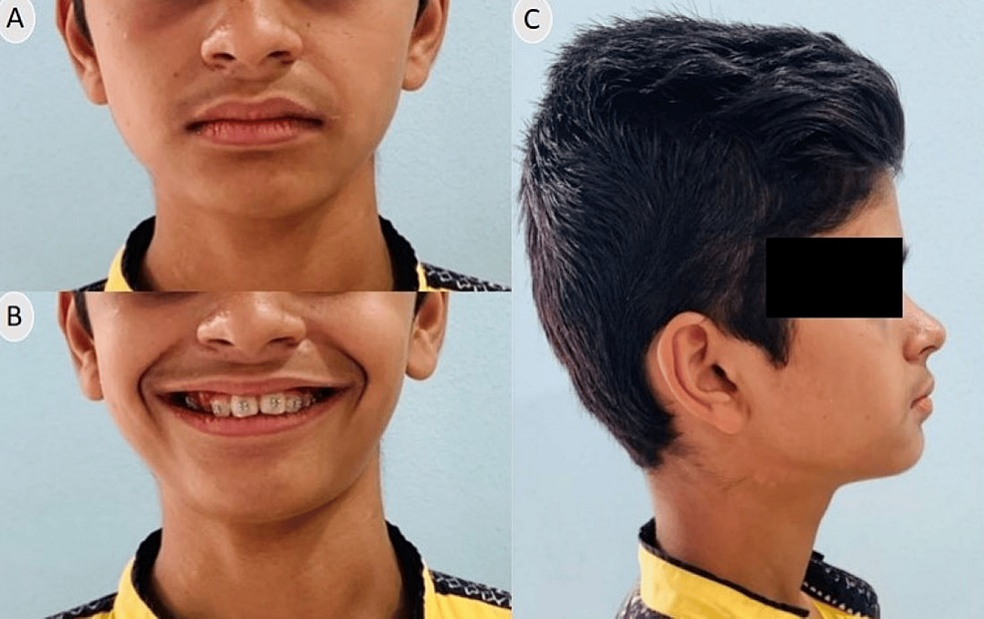
Extraoral photographs after phase II (Quad Block therapy) (A) Frontal; (B) Smiling; (C) Profile

Post-phase II treatment (Quad Block therapy) continued mandibular growth can be appreciated with a profile view of the patient (Figure 9C) along with a consonant smile (Figure 9B).

**Figure 10:**
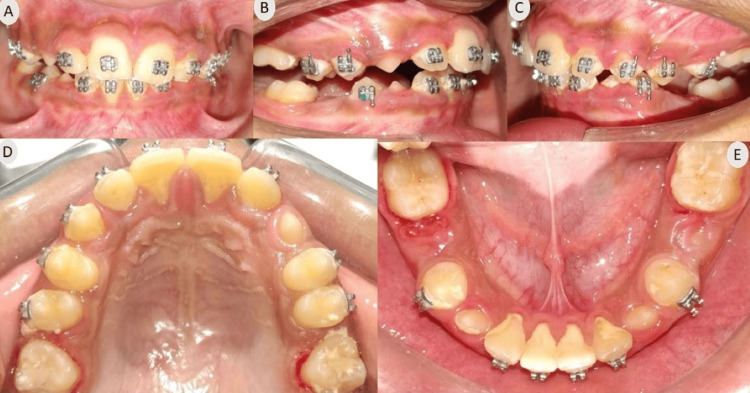
Intraoral photographs after phase II (Quad Block therapy) (A) Frontal view; (B) Right lateral view; (C) Left lateral view; (D) Maxillary occlusal view; (E) Mandibular occlusal view

Intraoral photographs post-phase II show maintained class I molar relation, erupting canines of all quadrants, and reduced overjet and overbite (Figure 10).

**Figure 11:**
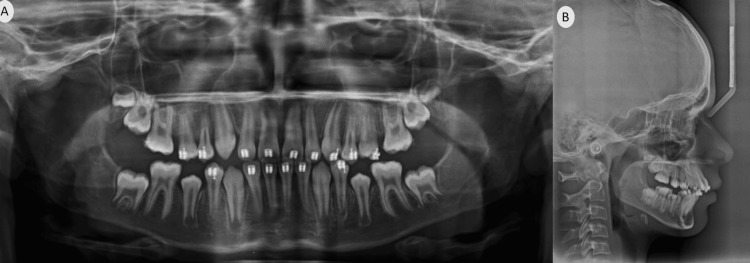
Post-Quad Block radiographs (A) Orthopantomogram; (B) Lateral cephalogram

The clinical objectives of the functional treatment phase were achieved successfully because of the patient's good compliance and follow-ups. This phase spanned a period of nine months. Notably, there was a retroclination of the upper incisors by 7° and a proclination of the lower incisors by 5°, which led to a reduction in the overjet (Figure 11, Table 1). The patient was instructed to activate the midline screw twice a week and was clinically assessed every four weeks.

In the subsequent phase of treatment, the Quad Block with fixed appliances was utilized. Quad block appliance is less bulky and is cemented which takes care of patient compliance. This appliance also allows the clinician to place brackets to achieve leveling and alignment at the same time, thus reducing the time of the treatment as well as giving visible results. The incisal opening of this appliance should not be more than 2 mm [5]. The objectives in this phase were to align and level the dentition while maintaining the achieved class I molar relationship from phase I. Overall, the treatment duration for both phases encompassed 15 months, with seven months dedicated to the Twin Block phase and eight months to the Quad Block with fixed appliance therapy.

## Discussion

The Twin Block functional appliance offers numerous established advantages, including its high patient tolerance, durability, ease of repair, and suitability for use in both permanent and mixed dentition stages. However, potential disadvantages such as lower incisors proclination and the risk of posterior open bites should be considered. In this case, successful achievement of treatment objectives can be largely attributed to the patient's commendable compliance. The patient's primary concern regarding an increased overjet was effectively addressed with the functional appliance, leading to enhanced confidence and minimized risk of upper incisor trauma [7].

The Twin Block appliance, a common functional orthodontic device, operates through a multifaceted mechanism of action aimed at correcting class II malocclusions by facilitating mandibular advancement and modifying jaw growth patterns. Its design incorporates upper and lower bite blocks that guide the lower jaw forward, promoting a more favorable occlusal relationship between the upper and lower teeth [7]. By encouraging mandibular repositioning, the appliance effectively reduces overjet and overbite, addressing the primary concerns associated with class II malocclusions. Additionally, the appliance promotes proper muscle function and coordination, harnessing the patient's natural oral functions such as chewing and swallowing to enhance treatment outcomes. Through consistent wear, patients engage in muscle training and adapt to the new jaw position, contributing to the long-term stability of the correction. Furthermore, the Twin Block appliance exerts orthopedic effects by stimulating mandibular growth and modifying jaw growth patterns, leading to favorable skeletal changes that support the overall correction of the malocclusion [7].

Baccetti et al. [8] observed that regardless of treatment timing, skeletal changes outweighed dental changes, particularly noting significant increases in mandibular length and height, particularly in older patients treated during the pubertal growth spurt. Notably, they found that the primary orthopedic effect was observed in the mandible, with no observed alterations in the sagittal position of the maxilla or vertical facial relationships. Similarly, Mills and McCulloch [9] attributed the majority of overjet reduction to mandibular skeletal changes. Lund and Sandler [10] corroborated these findings, emphasizing significant mandibular changes (as evidenced by the increased angle between cephalometric points S, N, and A (SNA)) with the Twin-block appliance while detecting no substantial maxillary skeletal changes. However, in contrast to Baccetti et al. [8] and Mills and McCulloch [9], they noted that dentoalveolar effects predominated over skeletal effects, with most of the overjet reduction attributed to dentoalveolar changes.

Regarding changes in incisors, most studies have consistently observed retroclination or retrusion of upper incisors, irrespective of the presence or absence of a labial bow. Jena et al. [11] suggested that the headgear effect of the labial bow, combined with its contact with the upper incisors during sleep, may contribute to maxillary incisor retroclination. Conversely, Toth and McNamara [12] proposed that retroclination or retrusion could be attributed to the pressure exerted by upper lip musculature during functional treatment, explaining retroclination even in studies where a labial bow was absent, as noted in the studies of Baccetti et al. [8], Mills and McCulloch [9], Illing et al. [13], Sidlauskas [14], and Toth and McNamara [12]. Overall, with the exception of Baccetti et al. [8], all studies have reported retroclination or retrusion of upper incisors, with more pronounced changes observed in studies utilizing an upper labial bow [9,13].

Similarly, all studies have documented proclination or protrusion of lower incisors following Twin Block treatment. This effect was observed even in studies where either a lower labial bow [9] or an acrylic extension covering the edges of the lower incisors [14] was employed. As highlighted by Jena et al. [11], mandibular protrusion leads to a mesial force application on the lower incisors, resulting in their proclination in the absence of lower lip pressure during Twin Block treatment.

Quad Block appliance is another functional appliance used for the correction of skeletal class II malocclusion [5]. In the Quad Block appliance, the occlusal inclined plane acts as a guiding mechanism for mandibular growth in downward and forward directions. Inclined planes are independently cemented. It is a purely tooth-borne fixed functional appliance that can be used simultaneously with fixed orthodontic appliances [5]. No adverse forces on the lower anteriors are present, so there is no need to control the lower anteriors, thus no anchorage loss. Fabrication is quick and trimming can be done chairside. Since the appliance is not continuous bilaterally, it is more justifiable to the tongue and its functions. Its main disadvantage is repeated dislodgement of bite blocks due to masticatory force and frequent recementation is thus required.

The selection of functional appliances depends on various factors, including patient age, compliance, as well as clinical preferences, familiarity, and available laboratory facilities.

Throughout treatment, the cephalometric analysis indicated favorable skeletal changes, with a reduction in the ANB angle towards a class I skeletal pattern. Notably, the upper incisor proclination reduced to 115° with respect to the sella nasion (SN) plane and 27° upper incisor to nasion point A (UI-NA), so they remain proclined. The lower incisors exhibited proclination by 5° (Table 1). The facial axis angle increased to 95°, which depicts the forward repositioning of the mandible. The Y-axis angle exhibited a 3° increase, which contributes to an increase in lower facial height (Table 1). Although there was no change in the mandibular plane angle and incisor mandibular plane angle (IMPA), vertical proportions increased during treatment, contributing to an improved profile, largely attributed to favorable growth patterns and possibly influenced by the functional appliance [15].

Comparison of pre-treatment and post-Quad block lateral cephalometric radiographs revealed favorable skeletal growth towards a class I pattern, with notable changes in maxillary and mandibular incisor positions, which can be appreciated with increased interincisal angle (120°) (Table 1). While functional appliances are primarily associated with dentoalveolar effects rather than long-term skeletal changes, they play a crucial role in facilitating subsequent fixed appliance treatment phases to achieve desirable outcomes.

Regarding soft tissue changes, a study employing three-dimensional optical surface laser scanning found clinically relevant improvements post-treatment with the Twin Block appliance [16]. In this case, the patient demonstrated improved profile aesthetics, which can be appreciated by nasomental angle post-Quad block (109°) and Ricketts E line (0/3), and diligent oral hygiene practices maintained gingival health. The patient expressed satisfaction with the outcome, although treatment is ongoing due to some permanent teeth yet to erupt. Regular reviews are planned during this phase to monitor progress effectively.

## Conclusions

The successful orthodontic treatment of the patient with class II malocclusion presented in this case report highlights the effectiveness of a phased approach involving functional and fixed appliances. Through careful planning and execution, the treatment objectives, including the reduction of overjet and overbite, establishment of a class I molar relationship, and enhancement of facial aesthetics, were achieved. The use of the Clark Twin Block appliance in the initial phase of treatment facilitated early correction of the malocclusion and provided a foundation for subsequent treatment with Quad Block and fixed appliances, which provided an advantage of initial corrections of dentition along with retaining advanced mandibular position achieved post-Twin Block. The patient's compliance and follow-up care were instrumental in achieving favorable treatment outcomes. The cephalometric analysis demonstrated favorable skeletal changes towards a class I pattern, accompanied by improvements in dental alignment and soft tissue aesthetics. Regular monitoring and adjustments throughout the treatment process ensured progress toward the desired outcomes.

Overall, this case underscores the importance of a comprehensive treatment approach tailored to the individual patient's needs and characteristics. By documenting the treatment process and outcomes, this case report contributes to the orthodontic literature and provides valuable insights for orthodontic practitioners managing similar clinical scenarios. The patient's satisfaction with the treatment outcome and ongoing progress serve as a testament to the efficacy of the treatment approach employed. Continued follow-up care will be essential to monitor long-term stability and address any emerging concerns, ensuring lasting oral health benefits for the patient.
